# Serological and Molecular Characterization of Hepatitis B Virus Infection in Gastric Cancer

**DOI:** 10.3389/fcimb.2022.894836

**Published:** 2022-05-03

**Authors:** Mengge Li, Shusheng Wu, Huiqin Luo, Jiayu Niu, Ying Yan, Yuan Fang, Lihong Ke, Wenju Chen, Huijun Xu, Huimin Li, Xiaoxiu Hu, Lulu Cao, Yaolin Chen, Hong Tu, Yifu He

**Affiliations:** ^1^ Department of Medical Oncology, The First Affiliated Hospital of University of Science and Technology of China (USTC), Division of Life Sciences and Medicine, University of Science and Technology of China, Hefei, China; ^2^ Organ Transplantation Center, The First Affiliated Hospital of Kunming Medical University, Kunming, China; ^3^ Department of Medical Oncology, Anhui Provincial Hospital Affiliated to Wannan Medical University, Wuhu, China; ^4^ State Key Laboratory of Oncogenes and Related Genes, Shanghai Cancer Institute, Renji Hospital, Shanghai Jiao Tong University School of Medicine, Shanghai, China

**Keywords:** gastric cancer, hepatitis B virus, infection, prognosis, mutation profiles

## Abstract

Hepatitis B virus (HBV) infection has been reported to be associated with gastric cancer (GC). Nonetheless, no study has revealed the role of HBV infection in the survival of patients with GC, and the mutation profiles of HBV-infected patients with GC have never been documented. Here, we performed an updated meta-analysis and found a significantly increased risk of GC in HBV-infected individuals (sOR, 1.29; 95% CI, 1.22-1.37). Furthermore, we observed that in the Anhui area, the rate of serum HBsAg positivity (OR, 1.62; 95% CI, 1.03-2.55) was significantly higher in GC patients than in controls. Moreover, our results showed that HBV-positive patients had significantly worse disease-free survival (HR, 1.98; 95% CI, 1.39-2.82) and overall survival (HR, 1.84; 95% CI, 1.19-2.85) than HBV-negative patients. The results of Cox proportional hazards regression proved that HBV infection was an independent adverse prognostic factor in GC. Furthermore, by performing targeted-NGS, we found unique mutation profiles in HBV-infected GC samples, including five frequently mutated protein-coding genes (*KMT2B*, *KMT2D*, *SOX1*, *FGF12*, and *TUBB2B*). Expression and survival analyses of these genes identified three novel candidate genes that may have potential roles in GC development. Gene Ontology enrichment analysis showed that the recurrent mutations in HBV-positive GC samples were related to cell proliferation, cell migration, and transcription. Taking together, our study proved that HBV infection is an independent prognostic factor in GC patients. The unique mutation profiles of HBV-infected patients with GC open a new research direction toward the underling mechanism between HBV infection and GC.

## Introduction

According to recent data from the International Agency for Research on Cancer, gastric cancer (GC) is the fifth most common cancer and the fourth most lethal cancer worldwide (Sung et al., 2021). In the past decades, the incidence rate, as well as the mortality rate of GC have increased worldwide, especially in East Asian countries (Tan et al., 2020). Surgical resection is recognized as the only potentially curative treatment, resulting in 5‐year overall survival (OS) rates of between 10% and 30% in patients with advanced GC. Established risk factors, including poor dietary habits, family history of cancers, and infections [e.g., Epstein-Barr virus (EBV), *Helicobacter pylori* (*H. pylori*), and human papillomavirus infections], have been suggested to contribute to the GC development (Yusefi et al., 2018). Recently, growing interests have focused on the association between hepatitis B virus (HBV) infection and GC (Tian et al., 2020; Hong et al., 2020; Yang et al., 2021; Wang et al., 2021).

Several epidemiological studies have found that the risk of GC was increased by chronic HBV infection. A meta‐analysis including 10 studies identified that the odds ratio (OR) of GC risk was 1.26 (95% confidence interval [CI], 1.08-1.47) in HBV carriers (Yang et al., 2021). However, several studies have shown conflicting results. The data from a case-control study in Iran failed to revealed the association between HBV infection and GC (OR, 2.13; 95% CI, 0.24-18.7) (Baghbanian et al., 2019). Another hospital registry-based case-control in Korea found HBV-infected individuals had an excess risk of GC but without statistical significance (OR, 1.03; 95% CI, 0.90-1.17) (An et al., 2018). Notably, although the association between HBV and GC is controversial worldwide, published studies involving Chinese patients, mostly from Southern China, showed a close connection between chronic HBV infection and GC development (Wei et al., 2015; Kamiza et al., 2016; Wei et al., 2017; Lu et al., 2018; Song et al., 2019; Tian et al., 2020; Wang et al., 2021). However, evidence on HBV infection in patients with GC from Central China is scarce (Tian et al., 2020). To date, the impact of HBV infection on the survival of GC patients has not been reported.

GC is a complex disease, the pathogenesis of which involves multiple genes and is highly related to variations in exon or intron groups. In the last two decades, after the introduction of next-generation sequencing (NGS), many driver genes involved in GC pathogenesis and several important signaling pathways have been identified, thus improving the understanding of the molecular mechanism of GC (Park et al., 2020; Businello et al., 2021; Gu et al., 2021; Sundar et al., 2021). For example, Wang et al. identified that *FGFR2* alteration was more frequent in poorly cohesive GC than in non-poorly cohesive GC, suggesting that *FGFR2* may be a potential therapeutic target for poorly cohesive GC (Wang et al., 2021). Badr et al. applied a computational biology approach using public databases and revealed a robust gene signature, which defines the pathology of *H. pylori*-induced GC (Badr et al., 2021). Han et al. found that *H. pylori* infection alters the repair of DNA double-strand breaks *via SNHG17* and promotes GC development (Han et al., 2020). Recently, Abe et al. found that lost *ARID1A* expression is a component of virus-host interactions in the pathogenesis of EBV-associated GC (Abe et al., 2021). However, no study investigating HBV as the infectious cause of GC has revealed the mutation profiles of HBV-infected patients with GC.

Approximately 248 million people worldwide are infected with HBV (Schweitzer et al., 2015). In China, although the overall HBV prevalence has declined in the past 20 years (Liu et al., 2016) owing to universal HBV vaccination programs, the absolute number of individuals with chronic HBV infection remains large because of the vast population size. Recently, the occurrence of GC has been rapidly increasing in most areas of China, making GC a major health burden. To reveal the relationship and the mechanism between chronic HBV infection and GC, we performed a comprehensive study involving a meta-analysis and a sero-epidemiological analysis to explore the link between HBV infection and the characteristics and prognosis of patients with GC. Moreover, to get insights into the underling mechanism of HBV-infected patients with GC, we first applied a targeted NGS method to reveal the mutational landscape and to identify potential therapeutic targets for HBV-infected patients with GC.

## Materials and Methods

### Literature Search

The MOOSE (Meta-analysis of Observational Studies in Epidemiology) guidelines were applied in performing the systematic literature review and analysis (Stroup et al., 2000). PubMed and EMBASE were searched using the following terms: “hepatitis B virus”, “hepatitis B”, “hepatitis virus”, or “HBV” combined with “gastric cancer”, “stomach cancer”, or “GC”. The end-of-search date was December 1, 2021. The titles and abstracts of the selected publications were reviewed to determine if an article was relevant to our study. Furthermore, we carefully checked the reference list of the article to identify additional relevant publications.

### Inclusion and Exclusion Criteria

Studies were eligible included in the meta-analysis if they met the following inclusion criteria: (1) focused on the relationship between HBV infection and GC; (2) defined and classified GC based on cytology or pathology, according to the World Health Organization classification system or the most widely used Working Formulation classification; (3) defined HBV infection as HBsAg positive or HBV DNA positive; (4) reported risk estimates [OR or hazard ratio (HR)] with 95% CIs or provided data that allow calculating these values; and (5) available in English. Any disagreements between the two authors on study inclusion or exclusion were resolved by a third author.

### Quality Assessment

We assessed the quality of each individual study using the Newcastle-Ottawa scale (NOS). After the assessment, studies were excluded from the analysis if they had low quality (quality score < 4).

### Data Analysis

The following data from the articles were extracted by two reviewers: title, first author’s name, year of publication, study design, country, control types, years of follow-up, total number and source of included participants, HBV status, GC diagnosis, matching and adjustments, and outcomes. Any ambiguities from the two reviewers were decided by the corresponding author.

### Patients and Samples

A total of 465 patients with cytologically or pathologically confirmed GC at the First Affiliated Hospital of the University of Science and Technology of China (USTC) between January 2014 and September 2021 were included in this study for the measurement of HBV seromarkers. Blood and tissue samples were collected before any treatment for the disease. Serum samples from 930 age-matched controls (patients admitted to the orthopedics and ear, nose, and throat departments of the hospital for conditions unrelated to HBV) were randomly obtained during the same period. In both the GC and control groups, none of the patients had any other types of primary cancer. From the GC group, we also identified 142 patients who underwent surgical resection (R0) to analyze the prognostic value of HBV infection. Paraffin-embedded tissue or blood samples from 16 patients with GC were also collected from the First Affiliated Hospital of USTC. The study was approved by the institutional ethics review committee of the First Affiliated Hospital of USTC and conducted according to the principles of the Declaration of Helsinki. We confirmed that all studies are conducted in accordance with relevant guidelines/regulations. The informed consent was waived by the approval of the Ethical Committee because of the retrospective nature (including the patients’ information stored in the hospital database and residual samples from previous clinical diagnoses).

### Follow-Up

The follow-up time was calculated from the date of surgery to the event date or the date of last contact. Follow-up continued until September 1, 2021. The primary endpoint was OS, which was calculated from the time of surgery to the time of death of any cause. The secondary endpoint was disease-free survival (DFS), which was calculated from the time of surgery to the first recurrence of the index cancer or to the date of all-cause death.

### Serologic Assay for HBV Infection

The expression level of HBsAg, antibodies to HBsAg (anti‐HBs), hepatitis B antigen (HBeAg), HBeAg (anti‐HBe), and hepatitis B core antigen (anti‐HBc) in the patients’ serum was tested using the Cobas e601 (Roche, Inc., Basel, Switzerland). Unfortunately, the HBV viral loads were not routinely determined.

### Targeted Gene Capture and NGS

Genomic DNA was extracted using the FlexiGene DNA kit (QIAGEN, Hilden, Germany) or GeneRead FFPE DNA kit (QIAGEN) following the manufacturer’s instructions. Customized gene capture chip-based NGS including 688 cancer-related genes was performed on the tissue or blood DNA samples. The detailed experimental procedure has been previously described (Li et al., 2020). The targeted chip was custom-designed and produced by BGI (Shenzhen, China). All regions were captured and sequenced using an MGISEQ-2000 platform (BGI). The sequencing coverage and quality statistics for each sample are summarized in **Table S1**. The genomic alterations, (e.g., copy number alterations, single nucleotide variants, small insertions and deletions, and mutational signatures) were analyzed by Shanghai Origingene Bio-pharm Technology (Shanghai, China).

### Bioinformatics Analysis

Mutational signatures were displayed with 96-context classification. A non-negative matrix factorization (NMF) approach was applied to estimate the 96-substitution pattern with 60 known Catalogue of Somatic Mutations in Cancer (COSMIC, v3.2) cancer signatures (https://cancer.sanger.ac.uk/signatures) and to infer their exposure contributions. It’s possible that some of the signatures extracted by NMF are very similar to signatures that are already known. Thus, to further interpret our results, a cosine similarity of more than 0.85 with an existing COSMIC signature was extracted. The detailed experimental procedure has been described in previous literature (Alexandrov et al., 2013). In this study, the UCSC Genome Browser (GRCh37/hg19) was used to annotating the mutated genes. Gene Ontology (GO) enrichment analysis of mutated genes was performed using the web-accessible functional annotation tool from the DAVID (version 6.8; https://david.ncifcrf.gov/). The Cancer Genome Atlas (TCGA) database (http://cancergenome.nih.gov) (Cancer Genome Atlas Research Network et al., 2013) was applied to analyze the mutated gene expression and survival data.

### Statistical Analysis

The pooled estimates of risk was used to estimate the absolute likelihood of HBV infection. A higher summary OR (sOR) corresponded to a high risk of HBV in patients with GC. Because the overall risk of GC is low, the relative risk in cohort studies mathematically approximates the OR in case-control studies, permitting the combination of case-control and cohort studies. Statistical heterogeneity among studies was calculated by I^2^ and Q estimates. If I^2^ was > 50%, a random-effects model was used. Otherwise, a fixed-effects model was used. The data from the included studies in our study were combined to generate the summary statistics using a random-effects model. We also conducted a meta-regression analysis to explore the influence of other study characteristics including study type, HBV prevalence area, and geographic region. Besides, a sensitivity analysis was conducted where one study at a time was omitted from the pooled estimate to assess whether individual studies substantially influenced the summary statistic. Begg’s funnel plots and Egger’s (*P* ≤ 0.05 was used as an indicator of publication bias) linear regression test were used to evaluate the publication bias. The details of the statistical analysis of meta-analysis data have been described in our previous study (Li et al., 2018). All statistical analyses were performed using Stata 10.1 software (Stata Corp., College Station, TX, USA). All *P* values were two-tailed, and *P* < 0.05 was considered statistically significant. We used the chi-square test or Fisher’s exact test to analyze the categorical variables that were expressed as proportions. To compare continuous variables, we used the Student’s t-test. Survival curve and multivariate analyses were performed as previously described (Wang et al., 2020). Statistical analysis was performed using SPSS software (version 22.0; SPSS Inc., Chicago, IL, USA) or GraphPad Prism (GraphPad Software, San Diego, CA, USA).

## Results

### Association of HBV Infection and GC in the Meta-Analysis

Firstly, an updated meta-analysis to explore the relationship between HBV and GC was conducted. The flow diagram of literature search is shown in the **Figure S1**. Four cohort studies (Sundquist et al., 2014; Kamiza et al., 2016; Song et al., 2019; Hong et al., 2020) and eight case-control studies (Wei et al., 2015; Wei et al., 2017; An et al., 2018; Lu et al., 2018; Baghbanian et al., 2019; Mahale et al., 2019; Tian et al., 2020; Wang et al., 2021) were finally enrolled in this analysis. The main characteristics of the included studies are summarized in **Table S2** and **S3**. According to the worldwide prevalence data of chronic HBV infection (Schweitzer et al., 2015), two studies were performed in low HBV epidemic regions, two were performed in lower-intermediate HBV epidemic regions, and eight were performed in higher-intermediate HBV epidemic regions.

**Figure 1:**
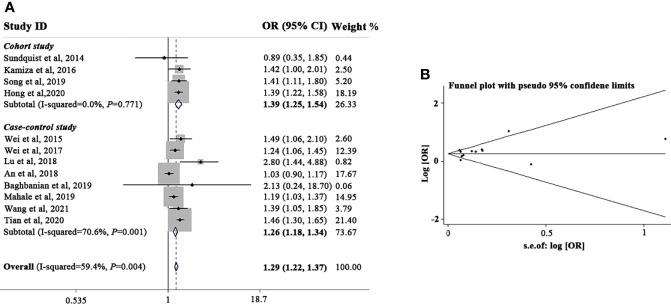
Meta-analysis and summary OR of the association between HBV infection and gastric cancer. **(A)** Summary OR of the association between HBV infection and gastric cancer. **(B)** Funnel plot of the studies included in the meta-analysis of the association between HBV infection and gastric cancer. HBV, hepatitis B virus; OR, odds ratio; CI, confidence interval.

We analyzed all the included studies and found that the sOR of GC in patients with HBV infection increased significantly (sOR, 1.29; 95% CI, 1.22-1.37; *P* < 0.001; **Figure 1A**). However, significant heterogeneity (I^2^) = 59.4%; *P* = 0.004) existed among these studies. Thus, according to HBV prevalence area, study type, and geographic location, we performed a meta-regression analysis to assess the potential resources of heterogeneity (**Table S4**). HBV prevalence area was found to be a main factor that affecting the correlation between HBV infection and GC (*P* = 0.010). Other variables had no significant effects on the heterogeneity.

Because the HBV prevalence area was found to have a substantial influence on the correlation between HBV infection and GC, we first stratified the analysis according to HBV prevalence areas. As illustrated in **Table S5**, HBV-infected patients from higher-intermediate HBV prevalent areas (sOR, 1.36; 95% CI, 1.23-1.52; *P* < 0.001) were prone to develop GC than those living in lower-intermediate HBV prevalent areas (sOR, 1.03; 95% CI, 0.91-1.18; *P* = 0.514) or low HBV prevalence areas (sOR, 1.18; 95% CI, 1.03-1.36; *P* = 0.021). When the analysis was restricted according to study design, we found that HBV infection increased the incidence of GC in both cohort studies (sOR, 1.38; 95% CI, 1.14-1.67; *P* < 0.001) and case-control studies (sOR, 1.19; 95% CI, 1.10-1.28; *P* < 0.001), indicating that different study types are equally effective in evaluating the correlation between HBV infection and GC. We also conducted a subset analysis according to geographic region. No significant association was found according to region (Asia vs. non-Asia; sOR, 1.22 vs. 1.18).

The funnel plot analysis of the included articles was shown in **Figure 1B**. We observed a good symmetrical distribution in the overall analysis and in all subset analyses, no substantial publication bias was showed by neither Begg’s test nor Egger’s test (Egger’s test, *P* = 0.065; Begg’s test, *P* = 0.371). By the sensitivity analysis, we found none studies significantly affected the overall results.

### HBV Infection in Patients With GC

As evidence on HBV infection in patients with GC from Central China is scarce, we first performed an epidemiological study in Anhui, China, involving 465 patients with GC and 930 age-matched controls (**Table 1**). The prevalence of HBsAg was highly associated with GC in both univariate analysis (OR, 1.91; 95% CI, 1.33-2.76; *P* = 0.001) and multivariate analysis (OR, 1.62; 95% CI, 1.03-2.55; *P* = 0.039; **Table 1**). Moreover, anti-HBc, a seromarker for past exposure to HBV, also showed a significant association with GC in both univariate analysis (OR, 1.43; 95% CI, 1.14-1.79; *P* = 0.002) and multivariate analysis (OR, 1.28; 95% CI, 1.00-1.64; *P* = 0.047; **Table 1**).

**Table 1:** Prevalence of HBV infection in gastric cancer patients and controls.

Variables	Control (n=930)	Gastric cancer (n=465)				
	no. (%)	no. (%)	Univariate analysis		Multivariate analysis	
			OR (95% CI)	*P*	OR (95%CI)	*P*
**Age**	61.42 ± 13.12	61.80 ± 10.17	NA	0.830	NA	NA
**Gender**						
Female	478 (51.40)	129 (27.74)	1		1	
Male	452 (48.60)	336 (72.26)	**2.75 (2.17-3.50)**	**0.000**	**2.64 (2.07-3.36)**	**0.000**
**HBsAg**						
Negative	862 (92.69)	404 (86.88)	1		1	
Positive	68 (7.31)	61 (13.12)	**1.91 (1.33-2.76)**	**0.001**	**1.62 (1.03-2.55)**	**0.039**
**Anti-HBs**						
Negative	436 (46.88)	213 (45.81)	1		1	
Positive	496 (53.12)	252 (54.19)	1.04(0.83-1.30)	0.719	1.02 (0.77-1.35)	0.909
**HBeAg**						
Negative	928 (99.78)	463 (99.57)	1		1	
Positive	2 (0.22)	2 (0.43)	1.00 (0.18-5.47)	0.999	0.84 (0.15-4.78)	0.839
**Anti-HBe**						
Negative	727 (63.98)	242 (52.47)	1		1	
Positive	335 (36.02)	221 (47.53)	**1.46 (1.17-1.83)**	**0.001**	1.23 (0.94-1.61)	0.139
**Anti-HBc**						
Negative	489 (52.58)	203 (43.66)	1		1	
Positive	441 (47.42)	262 (56.34)	**1.43 (1.14-1.79)**	**0.002**	**1.28 (1.00-1.64)**	**0.047**

CI, confidence interval; OR, odds ratio; Control, patients admitted to the orthopedics and ear, nose and throat departments of the hospital, unrelated to HBV; NA, not available.

Bold values indicate statistically significant difference.

**Figure 2:**
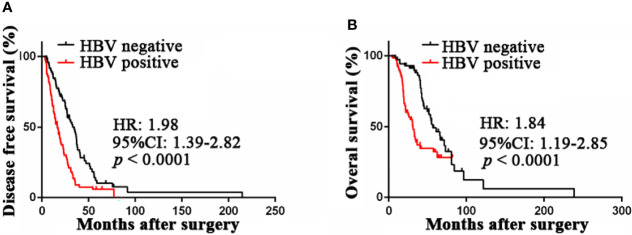
Prognostic value of HBV infection in patients with gastric cancer. **(A)** HBV-positive patients was associated with poor disease-free survival compared with HBV-negative patients by Kaplan-Meier curves analysis. **(B)** HBV-positive patients was associated with poor overall survival compared with HBV-negative patients by Kaplan-Meier curves analysis. HBV, hepatitis B virus; HBV-positive, positive for HBsAg or anti-HBs, anti-HBe, and anti-HBc; HBV-negative, negative for HBsAg, HBeAg, anti-HBe, anti-HBs, and anti-HBc.

To analyze the prognostic value of chronic HBV infection, seventy-one HBV-positive (HBsAg-positive for current infection or anti-HBs-, anti-HBc-, and anti-HBe-positive for previous infection) and 71 HBV-negative (HBsAg-, anti-HBs-, HBeAg-, anti-HBe-, and anti-HBc-negative) patients with GC were included. All patients underwent radical resection (R0) and selective chemotherapy. Univariate and multivariate logistic regression analyses of clinicopathological association reveled that patients with GC with HBV infection had a worse histological grade of GC (*P* < 0.05, **Table 2**). Interestingly, patients with HBV infection have a higher frequency of liver metastases after surgery (*P* < 0.05, **Table 2**) than patients without HBV infection. In addition, no significant differences in gender, age, body mass index, smoking, alcohol, and TNM (tumor, lymph node, and metastasis) stage were found. Moreover, our results revealed that GC patients with chronic HBV infection had significantly worse DFS (median: 16.67 vs. 33.00 months; HR, 1.98; 95% CI, 1.39-2.82; *P* < 0.001; **Figure 2A**) and OS (median: 30.85 vs. 56.87 months; HR, 1.84; 95% CI, 1.19-2.85; *P* < 0.001; **Figure 2B**) than those without HBV infection. We found that chronic HBV infection as an independent poor prognostic factor in GC development by the Cox proportional hazards regression (**Tables 3**, **4**). Besides, according to the multivariate survival analysis, we found that HBV-positive patients with GC had an approximately two times higher risk of disease progression (HR, 2.19; 95% CI, 1.49-3.21; *P* < 0.001) and death (HR, 2.24; 95% CI, 1.38-3.65; *P* = 0.001) than HBV-negative patients (**Table 4**).

**Table 2:** Univariate and multivariate logistic regression analyses for the associations between serum HBV positive and HBV negative postoperative gastric cancers.

Clinicopathologic features	HBV status		Univariate analysis		Multivariate analysis	
	Negative (n=71)	Positive (n=71)	*P*	OR (95% CI)	*P*	OR (95% CI)
**Gender**			0.573	1.24 (0.59-2.59)	0.749	0.87 (0.37-2.01)
**Male**	50	53				
**Female**	21	18				
**Age**			**0.03**	**2.10 (1.07-4.10)**	0.074	1.98 (0.94-4.17)
**<62**	41	28				
**≧62**	30	43				
**BMI**			0.430	0.66 (0.24-1.84)	0.510	0.83 (0.47-1.46)
**<18.5**	12	17				
**18.5-24**	43	39				
**>24**	16	15				
**Smoking**			0.350	1.83 (0.51-6.56)	0.854	1.19 (0.19-7.55)
**Never**	67	64				
**Ever**	4	7				
**Alcohol**			0.510	1.55 (0.42-5.73)	0.901	1.13 (0.17-7.52)
**Never**	67	65				
**Ever**	4	6				
**Liver metastasis**			**0.009**	**3.31 (1.35-8.09)**	**0.014**	**3.36 (1.27-8.88)**
**Positive**	8	21				
**Negative**	63	50				
**TNM**			0.052	2.48 (0.99-6.19)	0.120	2.21 (0.81-6.02)
**I-II**	17	8				
**III**	54	63				
**Histological grade**			**0.032**	**2.53 (1.08-5.92)**	**0.038**	**2.70 (1.06-6.93)**
**G1-G2**	19	9				
**G3**	52	62				

CI, confidence interval; OR, odds ratio; HBV positive, positive for HBsAg or anti-HBs, anti-HBe, and anti-HBc; HBV negative, negative for HBsAg, HBeAg, anti-HBe, anti-HBs, and anti-HBc; TNM, tumor, lymph node, metastasis; BMI, Body Mass Index.

Bold values indicate statistically significant difference.

**Table 3:** Univariate and multivariate Cox regression analyses for disease-free survival in patients with gastric cancer.

Clinicopathologic features	Total (n=142)	Median	Univariate analysis		Multivariate analysis		
		(months)	*P*	HR (95% CI)	*P*	HR (95% CI)	
**HBV statue**			**0.000**	**2.13 (1.55-3.21)**	**0.000**	**2.19 (1.49-3.21)**	
**Positive**	71	16.67					
**Negative**	71	33.00					
**Gender**			0.299	1.24 (0.83-1.86)	0.540	1.14 (0.75-1.75)	
**Male**	103	19.5					
**Female**	39	26.6					
**Age**			0.092	1.36 (0.95-1.94)	0.350	1.20 (o.82-1.78)	
**<62**	69	22.47					
**≧62**	73	18.7					
**TNM**			0.216	1.34 (0.84-2.14)	0.774	1.07 (0.66-1.77/	
**I-II**	25	26.7					
**III-IV**	117	19.5					
**Histological grade**			0.059	1.15 (0.95-1.76)	0.839	0.95 (0.61-1.50)	
**G1-G2**	28	27.27					
**G3**	114	20.77					

DFS, disease free survival; CI, confidence interval; HR, hazard ratios; HBV positive ,positive for HBsAg or anti-HBs, anti-HBe, and anti-HBc; HBV negative, negative for HBsAg, HBeAg, anti-HBe, anti-HBs, and anti-HBc; TNM, tumor, lymph node, metastasis.

Bold values indicate statistically significant difference.

**Table 4:** Univariate and multivariate Cox regression analyses for overall survival in patients with gastric cancers.

Clinicopathologic features	Total (n=142)	Median (months)	Univariate analysis		Multivariate analysis	
			*P*	HR (95% CI)	*P*	HR (95% CI)
**HBV statue**			**0.000**	**2.35 (1.548-3.73)**	**0.001**	**2.24 (1.38-3.65)**
**Positive**	71	30.85				
**Negative**	71	56.87				
**Gender**			0.460	1.21 (0.73-2.00)	0.834	1.06 (0.62-1.83)
**Male**	103	30.53				
**Female**	39	35.57				
**Age**			0.232	1.31 (0.84-2.04)	0.546	1.17 (0.70-1.94)
**<62**	69	42.03				
**≧62**	73	33.90				
**TNM**			0.593	1.16 (0.97-1.99)	0.750	0.91 (0.51-1.62)
**I-II**	25	43.63				
**III-IV**	117	33.07				
**Histological grade**			0.242	1.37 (0.81-2.33)	0.594	1.16 (0.66-2.04)
**G1-G2**	28	36.57				
** G3**	114	33.07				

OS, overall survival; CI, confidence interval; HR, hazard ratios; HBV positive, positive for HBsAg or anti-HBs, anti-HBe, and anti-HBc; HBV negative, negative for HBsAg, HBeAg, anti-HBe, anti-HBs, and anti-HBc; TNM, tumor, lymph node, metastasis.

Bold values indicate statistically significant difference.

### Genomic Landscape Between HBV-Positive and HBV-Negative Patients With GC

To study the underling mechanism by which HBV infection may participate GC progression, the genomic DNAs of seven HBV-positive and nine HBV-negative GC samples that were randomly picked from the above cases that underwent targeted-NGS. The clinical features of the involved patients are summarized in **Table S1**. All samples were microsatellite stable. For the seven HBV-positive GC samples, NGS identified 2446 and 689 somatic mutation sites in protein-coding and non-protein-coding regions, respectively (**Figure S2A** and **Table S6**). The mutation rate per Mb was 2.41 (range, 0.10-4.66). For the nine HBV-negative GC samples, a total of 776 and 658 somatic mutation sites in protein-coding and non-protein-coding regions were identified, respectively (**Figure S2A** and **Table S6**). The mutation rate per Mb was 3.95 (range, 0.10-8.96). The distributions of genetic variants in protein-coding regions between the two groups are presented in **Figures S2A, B**.

Thereafter, the number of single nucleotide variants were calculated in a matrix of ninty-six possible mutations that occurred in the tri-nucleotide context in patients with GC. Interestingly, in the HBV-positive group, the predominant mutation signature was signature B, with an increased proportion of C>A (G>T), C>T (G>A), and T>C (A>G) mutations (**Figures S2C, D**). Signature B is highly similar to COSMIC Signature 44, which is associated with defective DNA. We also found that signature A, which is associated with tobacco smoking, was the predominant mutation type in the HBV-negative group (**Figures S2C, D**).

**Figure 3:**
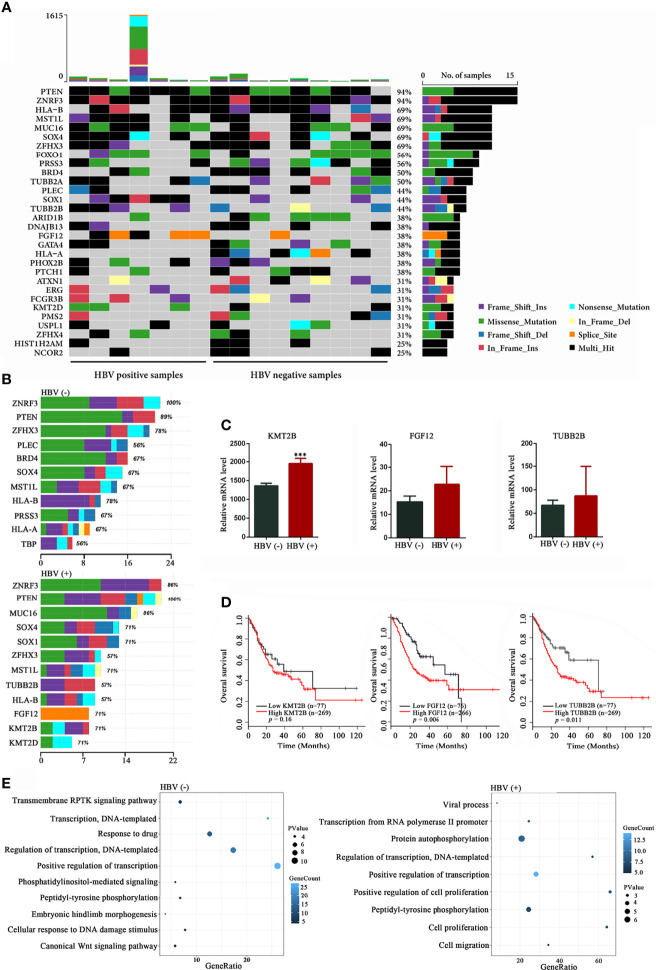
Mutational signatures in HBV-positive and HBV-negative samples. **(A)** Genetic profiles of seven HBV-positive and nine HBV-negative gastric cancer (GC) samples. **(B)** Comparison of the most frequently mutated genes between the HBV-positive and HBV-negative GC samples. **(C)** Expression analysis of *KMT2B*, *FGF12*, and *TUBB2B* in 32 pairs of GC and non‐tumor tissues from The Cancer Genome Atlas (TCGA) database. **(D)** Overall survival based on the expression levels of *KMT2B*, *FGF12*, and *TUBB2B* in 346 patients with GC from TCGA database by Kaplan-Meier curves analysis; **(E)** Gene Ontology annotation analysis of frequently mutated genes in GC (mutation frequency ≥ 2, *P* < 0.05). HBV, hepatitis B virus; HBV-positive, positive for HBsAg or anti-HBs, anti-HBe, and anti-HBc; HBV-negative, negative for HBsAg, HBeAg, anti-HBe, anti-HBs, and anti-HBc. ***P < 0.001.

The mutational landscape is summarized in **Figure 3**. Mutations in *PTEN* (94%), *ZNRF3* (94%), *HLA-B* (69%), *MUC16* (69%), *MST1L* (69%), *SOX4* (69%), *ZFHX3* (69%), *FOX1* (56%), *PRSS3* (56%), *BRD4* (50%), and *TUBB2A* (50%) were the most frequent alterations among the 16 patients with GC (**Figure 3A**). When comparing the two groups, we found that the frequencies of mutations in *KMT2B*, *SOX1*, and *FGF12* were significantly higher in HBV-positive group than in HBV-negative group (*P* = 0.035, **Figure S3** and **Figure 3B**), and the frequencies of mutations in *KMT2D* and *TUBB2B* showed an increasing mutated tendency in HBV-positive group than in the HBV-negative (*P* = 0.315, **Figure S3** and **Figure 3B**), suggesting that these mutated genes may exert a biological impact on HBV-infected patients with GC. Recently, several studies had revealed that *KMT2D* (Li et al., 2021; Numakura and Uozaki, 2021) and *SOX1* (Yin et al., 2020) were implicated in the initiation and progression of GC, whereas the other three frequently mutated genes have not been investigated. Thus, the mRNA expression levels of three frequently mutated genes in GC were analyzed by using TCGA datasets. Compared with paired non‐tumor tissues, *KMT2B* was found to be significantly upregulated in GC tissues (*P* < 0.05; **Figure 3C**). The expression levels of *FGF12* and *TUBB2B* showed an increasing trend in GC tissues than the paired non‐tumor tissues (*P* > 0. 05, **Figure 3C**). Besides, patients with high *FGF12* and *TUBB2B* expression levels had a shorter OS in the analysis of TCGA dataset (*P* < 0.05; **Figure 3D**). These results indicated that the frequently mutated genes may involve in GC development. We also performed GO enrichment analysis of recurrent mutations in the protein-coding genes (mutation frequency ≥ 2). We found that different HBV status performed distinct GO terms. GO analysis of the mutated genes in HBV-positive samples revealed terms related to cell proliferation, cell migration, and transcription (*P* < 0.05, **Figure 3E**). Meanwhile, the mutated genes identified from HBV-negative GC tissues were significantly involved in transcription and several signaling pathways (*P* < 0.05, **Figure 3E**).

## Discussion

Recently, HBV infection had been shown to be related with many extrahepatic cancers, and performed as a prognostic factor for the survival of patients (Li et al., 2020; Wang et al., 2021; Li et al., 2021). However, the relationship between HBV infection and GC significantly differed among different areas. Therefore, to study the association between HBV and GC, we first conducted an updated meta-analysis in this study, and found that HBV infection leads to a 1.29-fold increased risk of GC. Furthermore, we performed a large-scale epidemiological study in Anhui, in the central region of China. To our knowledge, no study has studied the effect of chronic HBV infection in patients with GC in Anhui. According to the cancer registration data in Anhui Province in 2015, the crude incidence and mortality rates of GC were significantly higher than the standardized rates in the Chinese population (incidence rate: 43.85/100,000 vs. 30.99/100,000; mortality rate: 31.22/100,000 vs. 21.33/100,000) (Lan et al., 2018). Thus, it is meaningful to study the pathogenic factors of GC, especially infectious agents such as HBV, in Anhui. Consistent with the meta-analysis, we found that the positive rate of HBsAg was significantly higher in patients with GC (OR, 1.62; 95% CI, 1.03-2.55) than in controls living in the same area.

Previously, a large body of retrospective studies investigated the prognostic of HBV on the survival of patients with extrahepatic cancers. Peng et al. reported that advanced non-small cell lung cancer patients with HBV infection had an adverse survival (Peng et al., 2015). Our previous study showed that patients with non-Hodgkin lymphoma with HBV infection had a significantly poor prognosis (Li et al., 2020). Zou and his colleges found that HBV infection was a favorable prognostic factor in the survival of patients with operable esophageal cancer. Nevertheless, the prognostic value of HBV infection for the survival after gastrectomy in patients with GC has not yet been evaluated. In this study, we found that HBV infection serves as an adverse prognostic factor in operable GC, and the incidence of liver metastasis after surgery was more frequent in patients with HBV infection than those without HBV infection. Moreover, HBV-positive GC patients had poorer DFS and OS than patients with an HBV-negative status, suggesting that HBV infection may perform as an adverse biomarker for predicting survival in GC patients. In the future, a prospective study is needed to furtherly evaluate the value of HBV infection in GC.

To date, little is known about the underlying mechanism of GC patients with HBV infection. The viral oncogenic hepatitis B virus X protein (HBx) has been shown to play a key role in the development of a variety of cancers. In the study by Cui (Cui et al., 2020), higher expression levels of HBx protein were found in GC tissues, and HBx-positive gastric epithelial cells demonstrated a higher nuclear-to-cytoplasmic ratio than HBx-negative cells, suggesting that chronic infection of HBV in gastric epithelial cells many induce carcinogenesis by viral oncoprotein HBx. Besides, increasing evidence showed local inflammation caused by the long-term chronic HBV infection might facilitate the cancerous transformation of gastric epithelial cells (34307172, 31199446). Recently, Song et al. speculated a critical role of impaired immune system between HBV infection and GC (31199446). Additionally, the integration of HBV DNA into the host genome has been considered as one of the mechanisms contributing to the development of HCC. In our previous studies, we found that HBV not only integrated in HCC tissues, but also in non-Hodgkin lymphoma (**) and intrahepatic cholangiocarcinoma (**). HBV insertion can result in host genome instability and *cis*-activation of the adjacent genes. Thus, we hypothesized that the integration of the viral genome into the host cellular DNA contribute to the altered expression of some cellular genes, which can lead to malignant transformation in gastric epithelial cells. Further investigations are still required.

It is widely accepted that recurrently mutated genes may have a role in HBV-related hepatocarcinogenesis (Guichard et al., 2012). Thus, to study the potential molecular mechanism of HBV infection that may promote the progression of GC, we comprehensively profiled the genomic features between HBV-positive and HBV-negative patients with GC. Compared with HBV-negative patients with GC, we found five frequently mutated genes (*KMT2B*, *KMT2D*, *SOX1*, *FGF12*, and *TUBB2B*) in HBV-positive patients with GC. *KMT2B* (lysine methyltransferase 2B), also known as *MLL2* (mixed lineage leukemia 2), belongs to the family of mammalian histone H3 lysine 4 methyltransferases and is known to be involved in the development of several cancers (Li et al., 2020; Klonou et al., 2021). Our previous study revealed that *KMT2B* was recurrently targeted by HBV DNA > 100 times in patients with hepatocellular carcinoma (Li et al., 2020), suggesting that *KMT2B* is a novel and critical regulator of hepatocellular carcinoma proliferation and metastasis (data not shown). Recently, He et al. found that *KMT2B* (22%) was one of the most frequently mutated genes in 262 patients with GC (He et al., 2021). To date, the effect of *KMT2B* in GC development has not been previously documented. In this study, the high frequency of *KMT2B* mutation in HBV-positive patients with GC, together with the finding that high expression level of *KMT2B* was related to shorter OS in patients with GC, highlights the need for further investigations into the biological function of *KMT2B* in HBV-related GC. In addition to *KMT2B*, we also found several other gene candidates on HBV‐related GC for further studies. *TUBB2B* and *FGF12* have been suggested to play vital functions in prostate cancer (Liotti et al., 2021), neuroblastoma (Zafar et al., 2021), and esophageal squamous cell carcinoma (Bhushan et al., 2017); however, their roles in HBV-related GC development have never been documented and merit further investigations.

Our study had several limitations. First, although a large sample was included, this study was a single-institution retrospective study, which may be a source of selection bias. Second, because of the lack of information on *H. pylori* and EBV infections in our study, the association between HBV and GC was not adjusted for these infections. Third, only seven HBV-positive and nine HBV-negative GC samples were available for NGS in our study. Therefore, further larger-scale investigations are needed to determine the exact pattern of mutations in HBV-related GC. Moreover, we used only the TCGA database to analyze the potential function of recurrently mutated genes in GC. Thus, we will use tumor and non-tumor samples from Chinese patients and performed functional studies to evaluate the role of HBV infection in GC development in the future.

In conclusion, the serological and molecular evidence from our study supporting the notion that HBV infection is significantly associated with GC and serves as an independent adverse prognostic factor. The information on HBV-related mutated genes provides new insights for investigating the mechanism of HBV-induced GC.

## Data Availability Statement

The datasets presented in this study can be found in online repositories. The names of the repository/repositories and accession number(s) can be found in the article/**Supplementary Material**.

## Ethics Statement

The studies involving human participants were reviewed and approved by institutional ethics review committee of the First Affiliated Hospital of USTC. The patients/participants provided their written informed consent to participate in this study.

## Author Contributions

YH was responsible for the study concept and design, analysis and interpretation of data, critical revision of the manuscript for important intellectual content, and obtaining funding. HT revised the manuscript. ML performed experiments, collected and analyzed data and wrote the manuscript. LK, YY, and WC performed the literature search and carried out the data inclusion and extraction. HLi, HX, and XH performed the quality assessment. SW, HLu, LC, and YC collected samples and analyzed data. SW, JN, and YF reviewed clinical data and performed statistical analyses. All authors read and approved the final manuscript.

## Funding

This study was funded by grants from the Fundamental Research Funds for the Central Universities (WK9110000172), Hefei Key Common Technology Research and Major Scientific and Technological Achievement Project (2021YL005), Natural Science Foundation of Anhui Province (1808085MH234), Anhui Province Key Research and Development Program Project (202104j07020044), and Health Commission of Anhui Province Scientific Research Project (AHWJ2021b105).

## Conflict of Interest

The authors declare that the research was conducted in the absence of any commercial or financial relationships that could be construed as a potential conflict of interest.

## Publisher’s Note

All claims expressed in this article are solely those of the authors and do not necessarily represent those of their affiliated organizations, or those of the publisher, the editors and the reviewers. Any product that may be evaluated in this article, or claim that may be made by its manufacturer, is not guaranteed or endorsed by the publisher.
