# Supplementary material for: Serological and Molecular Characterization of Hepatitis B Virus Infection in Gastric Cancer
Source: Front Cell Infect Microbiol. 2022 May 3;12:894836. doi: 10.3389/fcimb.2022.894836 (PMC9113707; doi:10.3389/fcimb.2022.894836)
Supplement: Supplementary file 1 [file DataSheet_1.docx]

Supplementary Material

##
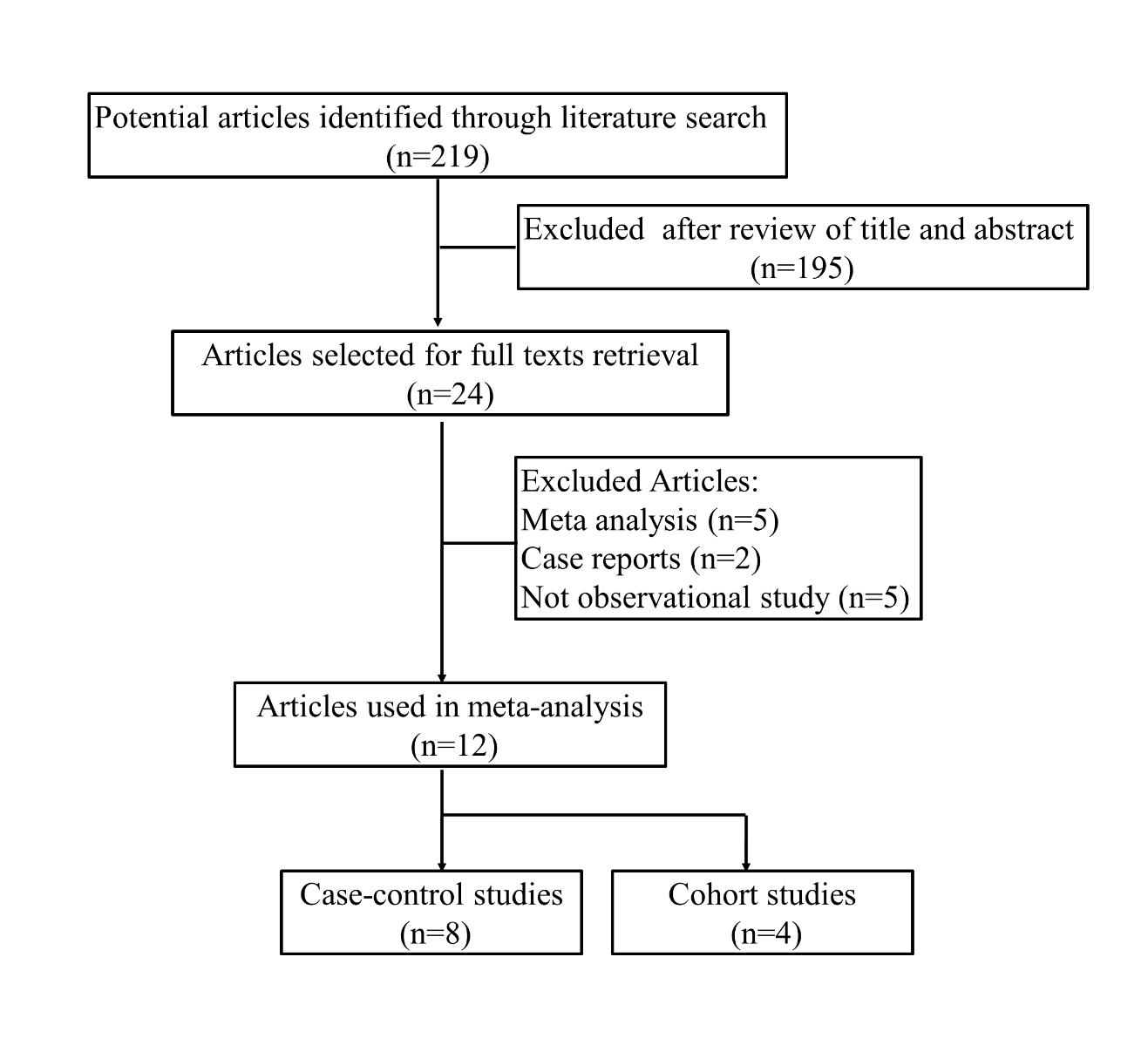
Supplementary Figures

## Fig. S1. Flowchart of the literature search


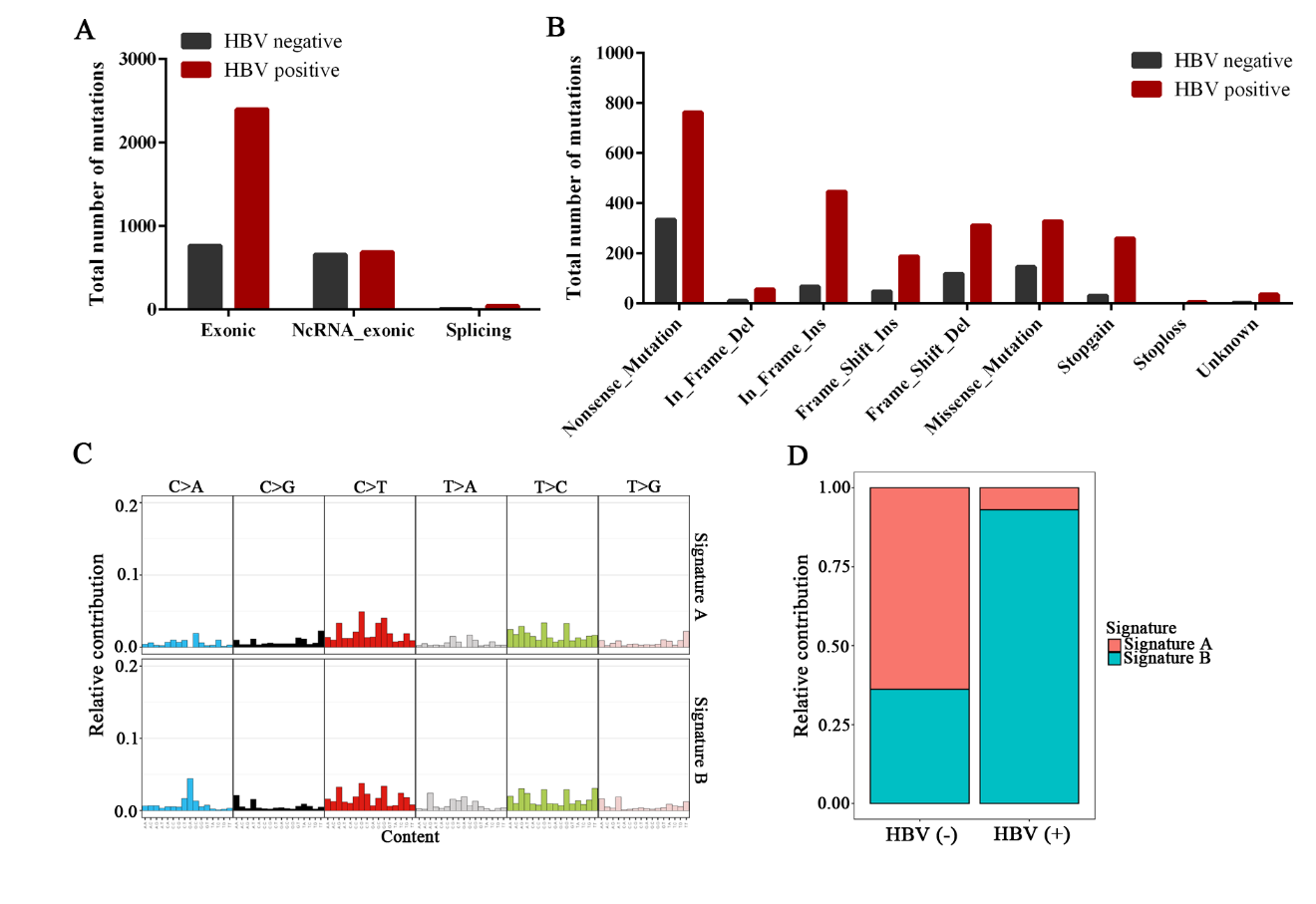


**Fig. S2. Mutation spectrum of 16 gastric cancer samples detected with targeted next-generation sequencing**

**(A)** Number of mutations in different genomic regions of the 16 gastric cancer (GC) samples. **(B)** Different mutation types in the region of protein-coding genes in the GC samples. **(C)** Lego plot showing the identification of two mutation signatures. **(D)** Distribution of the two mutation signatures in HBV-positive and HBV-negative patients with GC. HBV, hepatitis B virus; HBV-positive, positive for HBsAg or anti-HBs, anti-HBe, and anti-HBc; HBV-negative, negative for HBsAg, HBeAg, anti-HBe, anti-HBs, and anti-HBc.


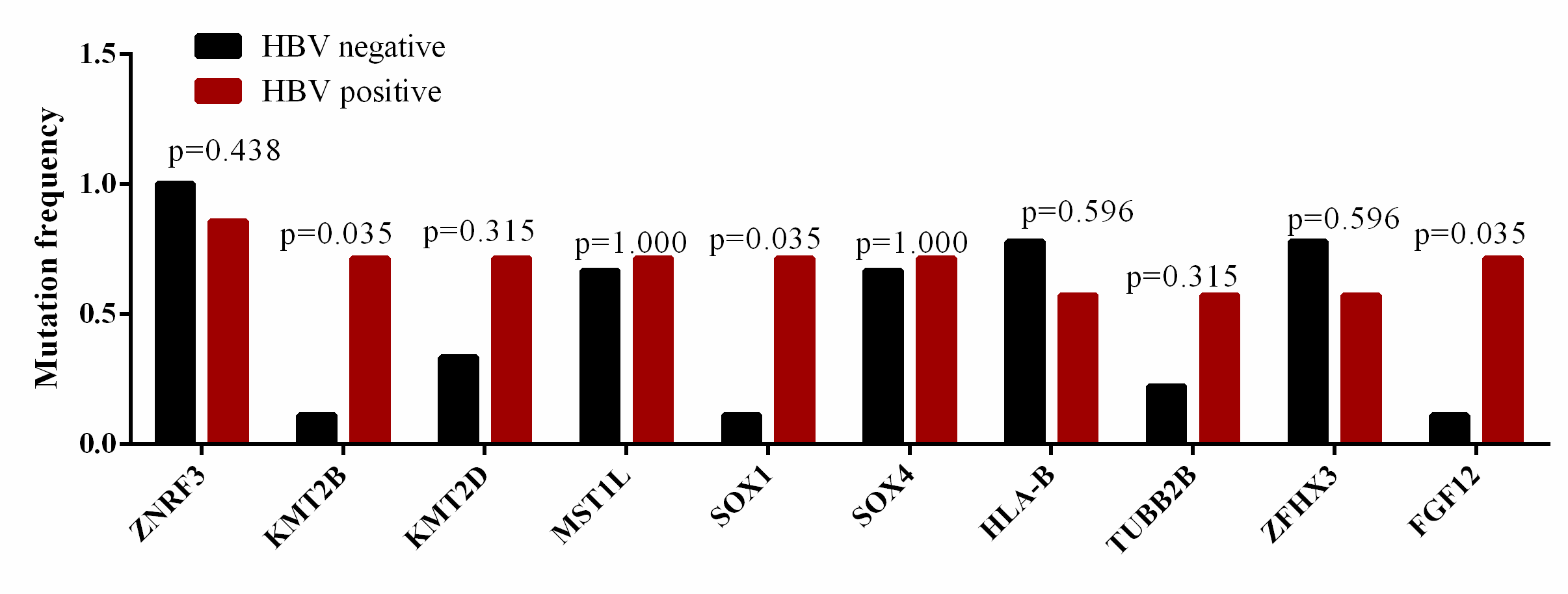
 **Fig. S3. The most frequent alterations of genes between HBV-positive and HBV-negative patients with GC.** HBV, hepatitis B virus; HBV-positive, positive for HBsAg or anti-HBs, anti-HBe, and anti-HBc; HBV-negative, negative for HBsAg, HBeAg, anti-HBe, anti-HBs, and anti-HBc

**Supplementary Tables**

**Table S2. Main characteristics of cohort studies included in the meta-analysis**

| Authors | Year | Source of  patients | HBV endemicity | Study  size | Source of study | Follow up period | Total person years | No. of patients with GC | NOS score | Language | Adjustments |
| --- | --- | --- | --- | --- | --- | --- | --- | --- | --- | --- | --- |
| Sundquist  et al. | 2014 | Sweden | low | 10,197 | The Swedish Hospital Discharge Register and  Outpatient Register | 1987-2000 | / | 7 | high | English | Age, gender, time period, occupation and region of residence |
| Kamiza et al | 2016 | China | higher-intermediate | 15,888 | The National  Health Insurance Research Database (NHIRD) | 2000-2011 | 940,100 | 2247 | high | English | Age and gender |
| Song et al | 2019 | China | higher-intermediate | 496,732 | The Qidong Cancer Registry | 2004-2008 | 4,400,000 | 78 | high | English | Age and gender |
| Hong et al | 2020 | Korea | lower-intermediate | 500,680 | NHIS-National Sample Cohort (NHIS-NSC) | 2003-2013 | 3,854,130 | 246 | high | English | Sex, BMI, smoking, drinking, income percentile, residential area, comorbidities |

HBV, hepatitis B virus; GC, gastric cancer; NOS, Newcastle-Ottawa Scale

**Table S3. Main characteristics of case-control studies included in the meta-analysis**

| Authors | Year | Source of  patients | HBV endemicity | Included period | No.  cases | Source of cases | No. controls | Source of controls | NOS score | Language | Adjustments |
| --- | --- | --- | --- | --- | --- | --- | --- | --- | --- | --- | --- |
| Wei  et al. | 2015 | China | higher-intermediate | 2007-2009 | 580 | Cancer Center of Sun Yat-sen University | 580 | Cancer Center of Sun Yat-sen University | high | English | Age, sex and year of diagnosis |
| Wei  et al. | 2017 | China | higher-intermediate | 2008-2014 | 2318 | Sun Yat-sen  University Cancer Center | 5,715 | Non-cancer population in Sun Yat-sen  University Cancer Center | high | English | Age, sex, year of diagnosis, smoking, drinking and family history of cancer. |
| Lu et al. | 2018 | China | higher-intermediate | 2012-2016 | 50 | Nanfang Hospital of Southern Medical  University, the People’s Hospital of Guangdong Province, and the Third  Affiliated Hospital, Sun Yat‒sen University | / | The general resident population aged 25–29 years in Guangdong province were obtained from the survey conducted in 2014–2015 | high | English | Age |
| An et al. | 2018 | Korea | lower-intermediate | 2007-2014 | 10,977 | Asan Medical Center | 118,891 | Asan Medical Center | high | English | Age, hypertension, diabetes, body mass index, alcohol consumption, smoking status, and cholesterol level in both genders |
| Baghbanian  et al. | 2019 | Iran | lower-intermediate | 2017-2018 | 728 | Yazd  Shaheed Sadoughi Hospital | 223 | Yazd  Shaheed Sadoughi Hospital | intermediate | English | Unadjustment |
| Mahale et al. | 2019 | The USA | low | 1993–2013 | 34,412 | SEER-Medicare linked database | 200,000 | SEER-Medicare linked database | high | English | Age, sex, race and  calendar year |
| Wang  et al. | 2021 | China | higher-intermediate | 2016-2017 | 1,326 | the West China Hospital, Sichuan University | 1,326 | the West China Hospital, Sichuan University | high | English | Age, sex,  BMI, smoking, alcohol drinking, diabetes mellitus, and family history of cancers according to tumor location before and after matching |
| Tian  et al | 2020 | China | higher-intermediate | 2008-2016 | 7,893 | the First Affiliated Hospital of  Nanjing Medical University | 11,361 | the First Affiliated Hospital of  Nanjing Medical University | high | English | Age and gender |

HBV, hepatitis B virus; GC, gastric cancer; NOS, Newcastle-Ottawa Scale

**Table S4. Meta-regression analysis of possible sources of heterogeneity.**

| Possible source of heterogeneity | Residual I^2^ (%) | *p* |
| --- | --- | --- |
| Study type | 57.43% | 0.821 |
| (case-control and cohort) |  |  |
| HBV prevalence area | 13.79% | 0.010 |
| (low, lower-intermediate and higher-intermediate) |  |  |
| Geographic region | 60.85% | 0.379 |
| (Asia and non-Asia) |  |  |

HBV, hepatitis B virus

**Table S5. Summary estimates for the association between hepatitis B virus infection and gastric cancer in subset analysis.**

| **Subgroup** | **No. of** | **sOR (95% CI)** | ***P*** | ***I*^2^ (%)** | ***P* for** |
| --- | --- | --- | --- | --- | --- |
|  | **studies** |  |  |  | **heterogeneity** |
| Study type |  |  |  |  |  |
| Case-control study | 8 | 1.19 (1.10-1.28) | <0.001 | 61.40 | 0.016 |
| Cohort study | 4 | 1.38 (1.14-1.67) | <0.001 | 0.00 | 0.571 |
| Geographic region |  |  |  |  |  |
| Non-Asia | 2 | 1.18 (1.03-1.36) | 0.021 | 0.00 | 0.500 |
| Asia | 10 | 1.22 (1.13-1.33) | 0.012 | 61.20 | <0.001 |
| HBV prevalence area |  |  |  |  |  |
| Low | 2 | 1.18 (1.03-1.36) | 0.021 | 0.00 | 0.500 |
| Lower-intermediate | 3 | 1.03 (0.91-1.18) | 0.514 | 0.00 | 0.630 |
| Higher-intermediate | 7 | 1.36 (1.23-1.52) | <0.001 | 30.10 | 0.209 |

HBV, hepatitis B virus
